# Supplementary material for: Subconfluent ARPE-19 Cells Display Mesenchymal Cell-State Characteristics and Behave like Fibroblasts, Rather Than Epithelial Cells, in Experimental HCMV Infection Studies
Source: Viruses. 2023 Dec 28;16(1):49. doi: 10.3390/v16010049 (PMC10821009; doi:10.3390/v16010049)
Supplement: Supplementary file 1 [file viruses-16-00049-s001.zip › Table.S2_qRT-PCR_primers_used_in_this_study.doc.pdf]

Table S2. *q*RT-PCR primers used in this study.

Primer name	Sequence	Source
VIM-fwd	GAGAACTTTGCCGTTGAAGC	(Mani et al., 2008)
VIM-rev	GCTTCCTGTAGGTGGCAATC	(Mani et al., 2008)
CDH1-fwd	TGTTCACCATTAACAGGAACAC	This study
CDH1-rev	GGGTATACGTAGGGAAACTCTC	This study
CDH2-fwd	TCAGTGAAGGAGTCAGCAG	This study
CDH2-rev	CTTCTGCCTTTGTAGGTGG	This study
CDH11-fwd	GCTGACTTGTGAATGGGAC	This study
CDH11-rev	TTGAGCTCATCACGTCAGG	This study
FN1-fwd	AAATGGCCAGATGATGAGC	This study
FN1-rev	TAACACGTTGCCTCATGAG	This study
FBN1-fwd	TAGGATGTGCAAAGATGAGG	This study
FBN1-rev	ATGAGGTTCTTGCATTCCA	This study
PPIA-fwd	AGCCAGGTACTTGGTGCTACAGTC	This study
PPIA-rev	TGCAGGTAGTCTGCGCCTTAAC	This study
C1orf116-fwd	CTCTGTCTCCATCTCTGCC	This study
C1orf116-rev	GCTATCACTCTCCACTGGG	This study
EPCAM-fwd	CGAGTGAGAACCTACTGGA	This study
EPCAM-rev	TGATCTCCTTCTGAAGTGCA	This study
GJB3-fwd	CTCATCATTGAGTTCCTCTTCC	This study
GJB3-rev	GCATATTGAAGCCATGCCA	This study
MARVELD3-fwd	AGAGATATCTGCCCTCGAC	This study
MARVELD3-rev	TCTGACTGGTAATATTCCACCTC	This study
ST14-fwd	CATGGAACATTGAGGTGCC	This study
ST14-rev	ATCTCCACGTAGTCCTTGG	This study
OVOL2-fwd	AAATCAAGTTCACCACAGGC	This study
OVOL2-rev	ACTTGAGGTGACGGTTCAG	This study
SNAI-fwd	TCTTTCCTCGTCAGGAAGC	This study
SNAI-rev	AGGTAAACTCTGGATTAGAGTCC	This study
