# Supplementary material for: Subconfluent ARPE-19 Cells Display Mesenchymal Cell-State Characteristics and Behave like Fibroblasts, Rather Than Epithelial Cells, in Experimental HCMV Infection Studies
Source: Viruses. 2023 Dec 28;16(1):49. doi: 10.3390/v16010049 (PMC10821009; doi:10.3390/v16010049)
Supplement: Supplementary file 1 [file viruses-16-00049-s001.zip › Table.S3_Antibodies_used_in_this_study.pdf]

Table S3. Antibodies used in this study.

Antibody	Dilution	Source	Identifier
Mouse monoclonal anti-E-Cadherin	1:500	BD Biosciences	Cat# 610181; RRID: AB_397580
Goat polyclonal anti-EPCAM	1:500	R & D Systems	Cat# AF960; RRID: AB_355745
Mouse monoclonal anti-β-tubulin	1:2000	The Developmental Studies Hybridoma Bank	Cat# E7; RRID: AB_528499
Mouse monoclonal anti-Vimentin	1:1000	The Developmental Studies Hybridoma Bank	Cat# AMF-17b; RRID: AB_528505
Goat polyclonal anti-Snail	1:500	R & D Systems	Cat# AF3639; RRID: AB_2191738
Rabbit polyclonal anti-OB-Cadherin	1:500	Cell Signaling Technology	Cat# 4442; RRID: AB_10547881
Mouse monoclonal anti-N-Cadherin	1:500	Cell Signaling Technology	Cat# 14215; RRID: AB_2798427
Mouse monoclonal anti-IE1 (clone 1B12)	1:1000	Shenk lab, Princeton University (Zhu et al., 1995)	N/A
Mouse monoclonal anti-UL99/pp28 (clone 10B4-29)	1:1000	Shenk lab, Princeton University (Silva et al., 2003)	N/A
Mouse monoclonal anti-UL83/pp65 (clone 8F5)	1:1000	Shenk lab, Princeton University (Nowak et al., 1984)	N/A
